# Expanding the Spectrum of Selective IgM Deficiency: From Infections to Immune Dysregulation

**DOI:** 10.3390/ijms26189003

**Published:** 2025-09-16

**Authors:** Rebecca Fumagalli, Francesco Saettini

**Affiliations:** 1 Dipartimento di Medicina e Chirurgia, Università degli Studi Milano-Bicocca, 20900 Monza, Italy; r.fumagalli22@campus.unimib.it; 2 Pediatria, Fondazione IRCCS San Gerardo dei Tintori, 20900 Monza, Italy; 3 Centro Tettamanti, Fondazione IRCCS San Gerardo dei Tintori, 20900 Monza, Italy

**Keywords:** IgM, hypogammaglobulinemia, primary immunodeficiencies, inborn errors of immunity, antibody deficiency

## Abstract

IgM plays a central role in early immune responses, yet the clinical significance of its deficiency remains poorly defined. Current diagnostic criteria focus on selective IgM deficiency (sIgMD), characterized by persistently low IgM concentrations and recurrent infections, potentially overlooking patients with isolated IgM deficiency and non-infectious manifestations. In this retrospective study, we analyzed a pediatric cohort with isolated IgM deficiency, irrespective of infectious history. Clinical features—including cytopenia, lymphoproliferation, autoimmunity, allergy, and inflammation—were similarly distributed in patients with and without infections. Importantly, 26% of patients received a molecular diagnosis consistent with inborn errors of immunity (IEIs), including several without recurrent infections. Longitudinal analysis revealed a dynamic course of IgM concentrations over time, allowing classification into chronic, intermittent, progressive, and resolved subtypes. These findings challenge the current definition of sIgMD, highlight the limitations of relying solely on infectious history, and suggest that isolated IgM deficiency may represent a broader and heterogeneous immunological phenotype. Molecular testing and extended follow-up may help identify underlying inborn errors of immunity and clarify long-term risks, even in patients initially lacking infectious complications. A redefinition of IgM deficiency is warranted.

## 1. Introduction

IEIs, formerly known as primary immunodeficiencies, represent a rapidly expanding group of monogenic disorders characterized by defects in immune development or function. These conditions predispose individuals to infections, autoimmunity, autoinflammation, lymphoproliferation, and malignancies. Advances in genomic technologies have identified over 500 distinct IEIs, revealing the complex interplay between immunity and host physiology [1,2].

Despite being recognized for decades, IgM deficiency remains rare and poorly understood, with an estimated prevalence ranging from 0.03% (complete IgM deficiency) in the general population to 0.07–2.1% in immunology clinics [3]. The IUIS defines sIgMD as absent serum IgM with preserved IgG and IgA concentrations [2]. ESID working criteria for clinical diagnosis of IEIs are even more stringent, requiring, along with IgM deficiency (<2 standard deviations [SD] below age-matched mean), preserved IgG and IgA, the presence of infections, normal concentrations of IgG subclasses and vaccination responses, and exclusion of T-cell defects [4]. Irrespective of the definition, secondary causes such as protein-losing enteropathies, nephrotic syndrome, or malignancy have to be excluded.

Clinical presentations are heterogeneous, ranging from asymptomatic individuals (IUIS definition) to patients with recurrent infections, allergies, autoimmunity, or malignancy [5]. However, the natural history and pathophysiological underpinnings of this condition remain elusive. Current definitions of sIgMD, requiring either undetectable IgM or the presence of infections, confer a narrow framework that may overlook patients with broader clinical phenotypes or evolving IgM concentrations.

This study aimed to reframe our understanding of isolated IgMD by analyzing pediatric patients with any form of IgMD, including those without infections and with variable trajectories over time. We hypothesized that IgMD represents a heterogeneous and dynamic condition, extending beyond infections to include immune dysregulation features.

## 2. Results

### 2.1. Patients’ Selection

Data were collected from 43 patients with reduced serum IgM concentrations and normal IgG and IgA. Four patients were excluded from further analysis due to either associated T-cell defects (one patient with DOCK8 deficiency) [6] or concurrent IgG subclass deficiencies (one individual with ADA2 deficiency [7], one with Claes-Jensen syndrome, and one with a clinical diagnosis of unclassified primary antibody deficiency).

### 2.2. Clinical and Immunological Characteristics of the sIgMD Cohort

Fifteen patients fulfilled criteria for sIgMD; of these, three met the ESID definition, and twelve were categorized as “possible sIgMD” [8,9]. The cohort was predominantly male (11/15, 73.3%), with a mean age at data collection of 11.7 years (range: 2–24) and a mean follow-up of 3.3 years (range: 0.3–9.4). Mean age at symptom onset was 5.0 years (range: 0–14.3), and decreased IgM concentrations were first documented at a mean age of 8.6 years (range: 0.9–17.8; Table 1). At the last follow-up visit, the age distribution was 1–2 years in 2/15 (13.3%), 2–6 years in 2/15 (13.3%), 6–12 years in 4/15 (26.7%), 12–18 years in 5/15 (33.3%), and >18 years in 2/15 (13.3%).

Infections were the most common initial clinical manifestation (9/15, 60.0%), followed by allergy (3/15, 20.0%), autoimmunity, cytopenia, and lymphoproliferation in one patient each (6.7%). No patients presented with inflammatory features at onset. However, the clinical phenotype broadened over time. At the time of IgM deficiency diagnosis, infections were present in 13/15 (86.7%) and cytopenia in 11/15 (73.3%). Allergies had increased to 5/15 (33.3%), while autoimmunity, lymphoproliferation, and autoinflammation were recorded in 3/15 each (20.0%). No patient remained asymptomatic. By the last follow-up, all patients had experienced infections, primarily of the respiratory tract. Recurrent HSV infections occurred in three cases. Two siblings developed hemophagocytic lymphohistiocytosis (HLH) following EBV infection. Cytopenias were documented in 14/15 (93.3%). Autoimmune manifestations were reported in six (40.0%)—including autoimmune thyroiditis, psoriasis, vitiligo (*n* = 2), and isolated autoantibody positivity (*n* = 2). Allergy was observed in five (33.3%) and inflammatory features in five (33.3%), including HLH (*n* = 2), vasculitis (*n* = 1), urticaria (*n* = 1), and recurrent unexplained fever (*n* = 1). Lymphoproliferation persisted in 3/15 patients (20.0%).

Six patients required treatment during follow-up: four received therapies for asthma and allergic symptoms (β2-agonists, inhaled corticosteroids, leukotriene receptor antagonists, and oral antihistamines), while two underwent HLH treatment protocols.

Genetic testing was performed in 9/15 patients (60.0%), yielding a molecular diagnosis in 5/9 (55.6%). Genetic testing was prompted by recurrent or severe infections (*n* = 5) and syndromic features (*n* = 4). Four patients (26.7%) met criteria for definitive IEIs per IUIS classification: one Jacobsen syndrome [10], one 22q11 deletion syndrome, and two X-linked lymphoproliferative syndrome type 2 (XLP2) [11]. One additional patient was diagnosed with MYT1L-related developmental delay–intellectual disability–obesity syndrome.

Immunophenotyping (Table 2) revealed reduced CD3^+^ and CD8^+^ T cells in 4/15 (26.7%) and reduced CD4^+^ and NK cells in 2/15 (13.3%). CD4^+^ subset analysis showed reduced recent thymic emigrants (RTEs) in 2/9 (22.2%), and altered naïve, terminally differentiated (TD), central memory (CM), or effector memory (EM) compartments in 1/10 each. CD8^+^ subset abnormalities included increased naïve (1/10), decreased CM (1/10), and decreased EM (2/10). CD19^+^ B cells were reduced in 1/14 patients (7.1%), excluding one patient who had received Rituximab. B-cell subset changes included increased naïve and TD B cells in 1/10 each. Overall, 9/15 patients (66.7%) had at least one lymphocyte subset abnormality. T-cell proliferation was normal in all tested patients (*n* = 4), though TCR repertoire analysis was abnormal in both patients tested. One patient had ANA positivity, and one had low C4 concentrations.

Serum IgM concentrations over time revealed dynamic trajectories (Table S1): 5/15 (33.3%) had persistently low IgM (chronic), 4/15 (26.7%) showed progressive decline, 5/15 (33.3%) had intermittent reductions, and one (6.7%) normalized. The diversity of clinical manifestations and longitudinal shifts in IgM concentrations—including the appearance of infections in patients initially infection-free—led us to expand our analysis beyond classical sIgMD.

### 2.3. Clinical and Immunological Characteristics of the IgMD Cohort

We identified 24 patients with IgM deficiency who did not present with infections (4 classified as “true” IgMD, 20 as “possible”). The majority were male (18/24, 64.3%). Mean age at symptom onset was 5.2 years (range: 0–14.3), mean age at diagnosis of decreased IgM was 7.6 years (range: 0.4–14.4), and mean age at last follow-up was 11.2 years (range: 2–18). Mean follow-up was 3 years (range 0.3–9.3; Table 3). At the last follow-up visit, patient ages were 1–2 years in 6/24 (25.0%), 2–6 years in 3/24 (12.5%), 6–12 years in 6/24 (25.0%), and 12–18 years in 9/24 (37.5%).

At disease onset, the most frequent symptom was cytopenia (16/24; 66.7%), followed by autoimmunity (4/24; 14.3%), and inflammatory manifestations (3/24; 10.7%). No patients initially presented with allergies or lymphoproliferation. At the time of IgM deficiency diagnosis, cytopenia was observed in 19/24 (67.9%), inflammation and autoimmunity in 4/24 each (14.2%). Allergy and lymphoproliferation were each noted in 2/24 (7.1%). By last follow-up, 23/24 patients (95.8%) had cytopenia. Autoimmune manifestations (e.g., alopecia, coeliac disease, autoimmune myelofibrosis [12], ANA positivity) were identified in 7/24 (29.2%), allergy in 4/24 (16.7%), and inflammatory features in 4/24 (16.7%), including vasculitis, urticaria, PFAPA-like symptoms, and recurrent fever in one patient each. Lymphoproliferation was observed in 2/24 (7.1%). No patients remained asymptomatic over time.

Seven patients received or were receiving treatment at last follow-up, including topical steroids for alopecia (one), antibiotic prophylaxis (one), allergy treatment (three), oral steroids for hemolytic anemia (one), oral steroids for recurrent fever episode (one), and levothyroxine for hypothyroidism (one).

Genetic testing was performed in 7/24 (29.2%), yielding a molecular diagnosis in 6/7 (85.7%). Testing was prompted by syndromic features (*n* = 4) and by recurrent fever, pancytopenia with bone marrow fibrosis, and chronic thrombocytopenia in one patient each. Two (8.3%) met IUIS criteria for IEIs (Jacobsen syndrome [10] and STXBP2 [12]). Other diagnoses included Down syndrome (*n* = 3) and von Willebrand disease (*n* = 1).

Immunophenotyping revealed abnormalities in T-, B-, and NK-cell subsets in the majority of patients (15/21, 71.4%; Table 4). Decreased CD3^+^, CD4^+^, and CD8^+^ T-cell counts were each observed in 7/21 (33.3%). CD4^+^ subset analyses (*n* = 5) showed reduced or increased RTEs (one each), and abnormalities in CM, EM, and TD subsets. CD8^+^ EM and CM subsets were decreased in one patient each. B-cell subset alterations included increased transitional or unswitched B cells (one each), reduced switched memory (1/5), and decreased TD cells (2/5). NK cells decreased in 3/21 (14.3%) and increased in 2/21 (9.5%). T-cell proliferation was preserved (*n* = 3). ANA positivity and low C3 were found in one patient each.

IgM concentrations evolved variably over time (Table S1): 14/24 (58.3%) had persistently low IgM (chronic), 4/24 (16.7%) had intermittent reductions, 4/24 (16.7%) normalized (resolved; median time for resolution = 58 months; range 20–94 months), and 2/24 (8.3%) exhibited progressive decline.

### 2.4. Comparison Between sIgMD and IgMD Groups

To explore the clinical relevance of the decreased IgM concentrations, we compared the SIgMD and IgMD groups at three key timepoints: onset, diagnosis, and last follow-up. Comparative analysis revealed no significant differences between sIgMD and IgMD groups in gender distribution (*p* > 0.99), age at symptom onset (*p* = 0.84), age at diagnosis (*p* = 0.57), age at last follow-up (*p* = 0.52), and length of follow-up (*p* = 0.74).

**Figure 1:**
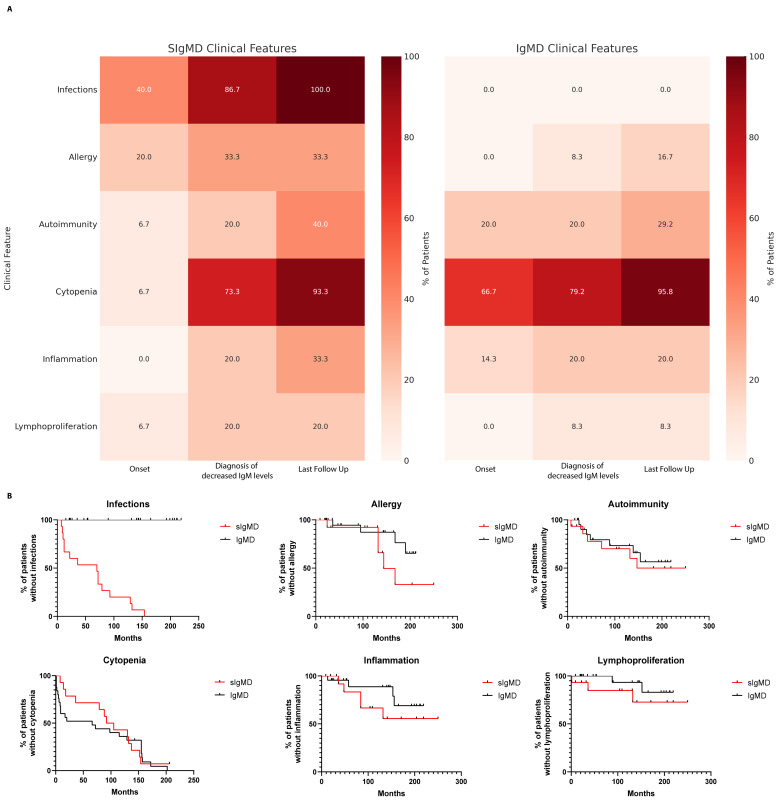
Comparison of clinical features in patients with selective IgM deficiency (sIgMD) and IgM deficiency without infections (IgMD). (**A**) Heatmap showing the distribution of major clinical features (infections, cytopenia, autoimmunity, allergy, inflammation, and lymphoproliferation) across timepoints (onset, diagnosis, and last follow-up) in sIgMD and IgMD patients. (**B**) Kaplan–Meier curves showing the cumulative incidence of clinical manifestations (autoimmunity, allergy, cytopenia, lymphoproliferation, and inflammation) over time in sIgMD and IgMD groups.

By definition, infections were absent in the IgMD group and significantly differed across all the timepoints (*p* < 0.0001). Excluding this feature, however, the prevalence of allergy, autoimmunity, autoinflammation, cytopenias, and lymphoproliferation was broadly comparable across groups and followed a similar temporal evolution. These findings underscore that non-infectious immune dysregulation is not limited to patients with sIgMD. Moreover, the rate of molecular diagnoses (33% vs. 25%) and IUIS-defined IEIs (26.7% vs. 8.3%) was similar, reinforcing that IgM deficiency may span a broader immunological spectrum than currently appreciated (Table 5 and Figure 1A).

Kaplan–Meier survival curves comparing the cumulative incidence of clinical manifestations over time revealed no statistically significant differences between groups (log-rank test, *p* > 0.05), indicating parallel trajectories of clinical progression (Figure 1B).

## 3. Discussion

The evolving landscape of IEIs is increasingly revealing a spectrum of phenotypes that extends beyond the classic presentation of severe, early-onset infections. Once largely defined by infectious susceptibility, many IEIs are now recognized to manifest with autoimmune disease, lymphoproliferation, allergy, autoinflammation, cytopenias, or subtle immunological abnormalities—even in the absence of infections [1,2]. Despite the recognition of immune dysregulation as a key feature of IEIs [13,14], the current ESID definition of sIgMD remains tightly linked to the presence of infections [4].

In this study, we examined a cohort of 39 patients with confirmed low IgM concentrations, of whom 15 met classical criteria for sIgMD. Although no gender bias was previously reported [15], we and others found male predominance [16,17,18,19]. The clinical phenotype of our cohort of sIgMD was consistent with prior reports: diagnosis in childhood, infections, and a variable degree of allergy and autoimmunity [16]. Importantly, however, several patients who initially lacked infections later developed this feature and increasing rates of autoimmune or allergic features were noticed. Moreover, cytopenias and lymphoproliferation were frequent and represent features not typically highlighted in current definitions. This broadening of the IgM deficiency phenotype over time supports the concept that IEIs are dynamic disorders, with manifestations that may shift or emerge over the disease course [20]. Our data confirm and extend this evolving view, underscoring immune dysregulation as a central element of IgM deficiency.

In our cohort, the rates of autoimmunity, allergy, cytopenia, and lymphoproliferation at last follow-up and the cumulative probability of these manifestations were remarkably similar between patients who fulfilled sIgMD criteria and those with IgMD but lacked early infections. This observation has key implications for clinical immunology.

First, infections, so far considered the hallmark of sIgMD, could not be considered as a core diagnostic criterion for sIgMD. Previous cohorts showed that up to 1/3 of patients may not show infections [16,17]. In our experience, patients without infections at onset or at diagnosis eventually developed infections during follow-up, while others manifested predominantly autoimmune or hematologic abnormalities. Some individuals with genetically confirmed IEIs also lacked infections entirely, despite clearly pathogenic molecular lesions and clinically significant immune dysregulation. This supports the concept of a shared immunological trajectory regardless of infectious history. These findings align with the increasing recognition that non-infectious phenotypes can dominate early disease in other IEIs [13,14], including monogenic syndromes with prominent thymic dysfunction (e.g., Jacobsen syndrome, 22q11.2 deletion syndrome) [10,21]. These findings argue against the inclusion of infections as a defining requirement for sIgMD, as this may delay recognition of patients with clinically relevant immune dysregulation who could benefit from monitoring and genetic testing. [22]

Second, our longitudinal analyses highlight the dynamic nature of IgM deficiency itself. IgM concentrations fluctuated over time, with patients transitioning from chronic or progressive deficiency to intermittent or resolved states. One patient in the sIgMD group and four in the IgMD group had normalization of IgM concentrations during follow-up, raising questions about the permanence of the diagnosis and underscoring the need for serial assessments. While the dynamic fluctuations of IgM concentrations (either in the progressive, intermittent, or resolved groups) may reflect biological trajectories of known or unknown molecular (either germline or somatic [6,23,24]) lesions, it raises practical concerns about follow-up duration. Such immunologic variability mirrors what is observed in other antibody deficiencies such as common variable immunodeficiency (CVID), where phenotypes can change over time [25].

Third, molecular testing in our cohort further supports that IgM deficiency may represent a shared immunophenotype across various monogenic and syndromic disorders. In total, 11/39 patients (28.2%) had a confirmed molecular diagnosis, including known IEIs such as Jacobsen syndrome, 22q11.2 deletion syndrome, and XLP2, as well as syndromes not yet officially recognized as IEIs (e.g., MYT1L, Down syndrome). Although some of these molecular diagnoses correspond to well-defined IUIS entities, we chose to include these patients in our analysis because they initially presented with isolated IgM deficiency, which was the entry criterion for cohort enrollment. Moreover, several IEIs, including those identified here, may first manifest through antibody abnormalities or immune dysregulation, with infections developing later during follow-up. Excluding such patients would have underestimated the overlap between IgM deficiency and monogenic IEIs and, importantly, would have biased the analysis toward milder phenotypes. IgM deficiency has been already reported in several chromosomal syndromes, such as Jacobsen syndrome [10], 22q11 deletion syndrome [26,27], and Down syndrome [17]. Strikingly, some of these patients with diagnosis of IEIs lacked recurrent infections and instead presented with autoimmunity or cytopenias, highlighting the limitations of a definition that centers on infection alone. Beyond these recognized IEIs, some patients carried diagnoses not classically considered IEIs—such as Down syndrome or MYTL—but with emerging literature pointing to immune dysregulation, reduced thymic output, and altered B- and T-cell compartments, these suggest that the boundaries of clinical immunology are broader than currently appreciated [28,29,30,31].

IgM deficiency should be interpreted as a possible early marker of immune dysregulation, even in the absence of infections. As such, patients with isolated IgM deficiency—regardless of whether they fulfill sIgMD criteria—deserve careful immunologic characterization and follow-up, including surveillance for cytopenias, autoimmunity, allergy, and lymphoproliferation, as well as consideration of molecular testing, especially when features evolve over time. Immunophenotyping of lymphocyte subsets in our cohort revealed abnormalities in the majority of patients, reinforcing the notion that IgM deficiency rarely exists in immunologic isolation [16]. For patients in whom IgM deficiency resolves spontaneously, we recommend continued follow-up, as normalization does not exclude later relapse (intermittent IgMD) or the emergence of other immune-mediated complications. Moreover, patients with “resolved” IgM deficiency may still harbor molecular defects that predispose them to late-onset manifestations, a phenomenon increasingly observed in adult-onset IEIs. As our understanding of immune dysregulation continues to grow, and as sequencing technologies become more accessible, it is likely that many patients with “isolated” IgM deficiency will ultimately be reclassified within the expanding universe of genetically defined IEIs.

This study has several limitations. First, its retrospective design limits the consistency of data collection as clinical and immunological evaluations were performed at the discretion of treating physicians. Second, the cohort is pediatric, with relatively short follow-up, limiting assessment of long-term trajectories. As our data suggest that IgM deficiency can evolve over time, longer prospective studies with extended follow-up are needed. Third, not all patients underwent molecular testing, extended immunophenotyping or functional immune assessments, potentially underestimating the prevalence of IEIs or immune dysfunction. Dual molecular diagnoses or multilocus genomic variations have been identified in up to 5% of patients undergoing whole-exome sequencing [32] and in approximately 10% of those investigated for IEIs [33]. As WES was not systematically performed in our cohort, we cannot rule out the possibility that some individuals may harbor additional pathogenic variants that contribute to their immunological phenotype [34]. Finally, including IUIS-defined IEIs may inflate the rate of genetic diagnoses in our cohort, and the relatively small cohort further limits generalizability and the depth of statistical analyses.

## 4. Material and Methods

### 4.1. Patients

A retrospective observational study was performed in pediatric patients (age < 18 years at the time of the first evaluation) followed at the Pediatric Immunology–Hematology Outpatient Clinic, IRCCS San Gerardo dei Tintori, Monza, Italy. Informed consent was obtained from patients, parents, or legal guardians. Patients with IgMD (IgM concentration < 2SD of the lower limit compared with those of healthy, age-matched control subjects) and normal serum IgG and IgA concentrations were included in the study, confirmed with at least two measurements with a 6-week interval. Exclusion criteria included concomitant IgG or IgA deficiency at diagnosis or secondary hypogammaglobulinemia (caused by infections, drugs, malignancies, protein-losing enteropathy, nephrotic syndrome, and thymoma).

Clinical and laboratory data were collected from medical records. When available, laboratory evaluation included complete blood count with differential, immunoglobulin concentrations (IgG, IgM, IgA, IgE, and IgG subclasses), antinuclear antibodies (ANA), C3 and C4, and immune response to vaccine protein antigens (tetanus and diphtheria). The last available flow cytometry for absolute counts of T (CD3^+^, CD3^+^CD4^+^, CD3^+^CD8^+^)-, B (CD19^+^)-, and NK (CD3^−^CD16^+^CD56^+^)-cells was compared with age-matched reference ranges. In a subgroup of patients, T- and B-cell subsets were investigated. Additional testing, such as celiac disease screening and other autoantibody investigations (i.e., thyroid autoimmunity), was performed when deemed appropriate (i.e., in case of abnormal thyroid function or gastrointestinal symptoms). History of infections, cytopenia, polyclonal lymphoproliferation, autoimmunity, allergy, inflammatory symptoms, and treatments were annotated.

Molecular analyses were performed on a patient basis at the clinician’s discretion.

### 4.2. Definitions of IgM Deficiency Phenotypes

sIgMD was defined in the presence of infections and a serum IgM concentration repeatedly below 2 SD of age-matched reference range with normal concentrations of serum IgA, IgG, normal vaccination responses (at the end of vaccination schedule), absence of T-cell defects, absence of IgG subclasses deficiency, and absence of causative external factors [4]. In the absence of a history of infections, patients were classified with IgM deficiency (IgMD). Irrespective of history of infections, patients were classified “truly”, if the aforementioned criteria were completely fulfilled, or “possible”, if incomplete data prevented confirmation of all required criteria [8,9].

To characterize the longitudinal patterns, patients were classified based on serial serum IgM measurements performed during clinical follow-up, according to age-matched reference ranges. After diagnosis of IgM deficiency, patients were categorized into the following groups:

- Chronic IgM deficiency: Persistently low serum IgM concentrations (<2SD) in all available determinations, without any evidence of normalization throughout follow-up.
- Intermittent IgM deficiency: Initial diagnosis of IgM deficiency followed by at least one single subsequent measurement within the normal range, without sustained normalization.
- Progressive IgM deficiency: Documentation of previously normal serum IgM concentrations that progressively declined to values below <2SD over time.
- Resolved IgM deficiency: Documented normalization of serum IgM concentrations on at least two separate measurements following an initial diagnosis of IgM deficiency.

### 4.3. Statistics

Descriptive statistics were used to summarize demographic, clinical, and immunological features. Continuous variables were expressed as means with ranges, and categorical variables as counts and percentages. Comparisons between groups (e.g., sIgMD vs. IgMD) were performed using the Chi-square test or Fisher’s exact test for categorical variables, and Student’s t-test for continuous variables, as appropriate. A *p*-value < 0.05 was considered statistically significant. Kaplan–Meier survival curves were generated to compare the cumulative incidence of clinical manifestations over time in patients with sIgMD versus those with IgMD. Separate analyses were performed for each major clinical domain, including autoimmunity, allergy, cytopenia, lymphoproliferation, and inflammation. All analyses were conducted using GraphPad Prism 8.0.2.

## 5. Conclusions

In conclusion, our data suggest that SIgMD is part of a broader, evolving immunological phenotype. Infections, while common, may not be considered a necessary criterion for diagnosis. The dynamic trajectory of IgM concentrations, the high frequency of immune dysregulation, and the substantial proportion of patients with underlying monogenic or chromosomal defects underscore the need for comprehensive and longitudinal evaluation. We advocate for a broader and more inclusive approach to diagnosing and managing IgM deficiency—one that recognizes the diversity and temporal evolution of clinical features and aligns with the current understanding of IEIs.

## Figures and Tables

**Table 1:** Demographic and clinical characteristics of patients with selective IgM Deficiency.

	Onset	Diagnosis of Decreased IgM Concentrations	Last Follow-Up
Gender, male			11/15 (73.3)
Age, years	5.0 (0–14.3)	8.6 (0.9–17.8)	11.7 (2–24)
Follow-up, years			3.3 (0.3–9.4)
Infections	9/15 (60.0)	13/15 (86.7)	15/15 (100)
Allergy	3/15 (20.0)	5/15 (33.3)	5/15 (33.3)
Autoimmunity	1/15 (6.7)	3/15 (20.0)	6/15 (40.0)
Inflammation	0/15 (0.0)	3/15 (20.0)	5/15 (33.3)
Cytopenia	1/15 (6.7)	11/15 (73.3)	14/15 (93.3)
Lymphoproliferation	1/15 (6.7)	3/15 (33.3)	3/15 (20.0)
Treatment			6/15 (40.0)
Molecular diagnosis			5/15 (33.3)
IEI diagnosis			4/15 (26.7)
IgM concentrations over time			
Chronic			5/15 (33.3)
Progressive			4/15 (26.7)
Intermittent			5/15 (33.3)
Resolved			1/15 (6.7)

**Table 2:** Lymphocyte subsets in the selective IgM Deficiency cohort.

	Low	Normal	High
CD3^+^	4/15 (26.7)	11/15 (73.3)	0/15 (0.0)
CD4^+^	2/15 (13.3)	13/15 (86.7)	0/15 (0.0)
Recent thymic emigrants	2/9 (22.2)	7/9 (77.8)	0/9 (0.0)
Naïve	1/10 (10.0)	9/10 (90.0)	0 (0.0)
Central memory	0/10 (0.0)	9/10 (90.0)	1/10 (10.0)
Effector memory	0/10 (0.0)	9/10 (90.0)	1/10 (10.0)
Terminally differentiated	1/10 (10.0)	9/10 (90.0)	0/10 (0.0)
CD8^+^	4/15 (26.7)	11/15 (73.3)	0/15 (0.0)
Naïve	0/10 (0.0)	9/10 (90.0)	1/10 (10.0)
Central memory	1/10 (10.0)	9/10 (90.0)	0/10 (0.0)
Effector memory	2/10 (20.0)	8/10 (80.0)	0/10 (0.0)
Terminally differentiated	0/10 (0.0)	10/10 (100)	0/10 (0.0)
CD19^+^	1/14 (7.1)	13/14 (92.9)	0/10 (0.0)
Transitional	0/9 (0.0)	9/9 (100)	0/9 (0.0)
Naïve	0/10 (0.0)	9/10 (90.0)	1/10 (10.0)
CD19hiCD21lo	0/9 (0.0)	9/9 (100)	0/9 (0.0)
Unswitched	0/10 (0.0)	10/10 (100)	0/10 (0.0)
Switched	0/10 (0.0)	10/10 (100)	0/10 (0.0)
Terminally differentiated	0/10 (0.0)	9/10 (90.0)	1/10 (10.0)
CD3^−^CD16^+^CD56^+^	2/15 (13.3)	13/15 (86.7)	0/15 (0.0)

**Table 3:** Demographic and clinical characteristics of patients with IgM deficiency without infections.

	Onset	Diagnosis of Decreased IgM Concentrations	Last Follow-Up
Gender, male			18/24
Age, years	5.2 (0–14.3)	7.6 (0.4–14.4)	11.2 (2–18)
Follow-up, years			3.0 (0.3–9.3)
Infections	0/24 (0.0)	0/24 (0.0)	0/24 (0.0)
Allergy	0/24 (0.0)	2/24 (7.1)	4/24 (14.3)
Autoimmunity	4/24 (14.3)	4/24 (14.3)	7/24 (29.2)
Inflammation	3/24 (10.7)	4/24 (14.3)	4/24 (14.3)
Cytopenia	16/24 (66.7)	19/24 (67.9)	23/24 (95.8)
Lymphoproliferation	0/24 (0.0)	2/24 (7.1)	2/24 (7.1)
Treatment			7/24 (29.2)
Molecular diagnosis			6/24 (25.0)
IEI diagnosis			2/24 (7.1)
IgM concentrations over time			
Chronic			14/24 (58.3)
Progressive			2/24 (7.1)
Intermittent			4/24 (14.3)
Resolved			4/24 (14.3)

**Table 4:** Lymphocyte subsets in patients with IgM deficiency without infections.

	Low	Normal	High
CD3^+^	7/21 (33.3)	13/21 (61.9)	1 (4.8)
CD4^+^	7/21 (33.3)	13/21 (61.9)	1 (4.8)
Recent thymic emigrants	1/5 (20.0)	3/5 (60.0)	1/5 (20.0)
Naïve	2/5 (40.0)	3/5 (60.0)	0 (0.0)
Central memory	1/5 (20.0)	4/5 (80.0)	0/5 (0.0)
Effector memory	1/5 (20.0)	4/5 (80.0)	0/5 (0.0)
Terminally differentiated	0/5 (0.0)	4/5 (80.0)	1/5 (20.0)
CD8^+^	7/21 (33.3)	13/21 (61.9)	0/21 (0.0)
Naïve	0/5 (0.0)	5/5 (100)	0/5 (0.0)
Central memory	1/5 (20.0)	4/5 (80.0)	0/5 (0.0)
Effector memory	1/5 (20.0)	4/5 (80.0)	0/5 (0.0)
Terminally differentiated	0/5 (0.0)	4/5 (80.0)	1/5 (20.0)
CD19^+^	5/21 (23.8)	14/21 (66.7)	2/21 (9.5)
Transitional	1/5 (20.0)	4/5 (80.0)	0/5 (0.0)
Naïve	0/5 (0.0)	5/5 (100)	0/5 (0.0)
CD19hiCD21lo	0/5 (0.0)	4/5 (80.0)	1/5 (20.0)
Unswitched	0/5 (0.0)	4/5 (80.0)	1/5 (20.0)
Switched	2/5 (40.0)	2/5 (40.0)	1/5 (20.0)
Terminally differentiated	0/5 (0.0)	3/5 (60.0)	2/5 (40.0)
CD3^−^CD16^+^CD56^+^	3/21 (14.3)	16/21 (76.2)	2/21 (9.5)

**Table 5:** Comparison between sIgMD and IgMD groups.

	Onset		Diagnosis of Decreased IgM Concentrations		Last Follow-Up	
Gender, male					11/15 vs. 18/24	>0.99
Age, years	5.0 vs. 5.2	0.84	8.6 vs. 7.6	0.57	12.5 vs. 11.2	0.52
Follow-up, years					3.2 vs. 3.0	0.74
Infections	6/15 vs. 0/24	<0.0001	13/15 vs. 0/24	<0.0001	15/15 vs. 0/24	<0.0001
Allergy	3/15 vs. 0/24	0.05	5/15 vs. 2/24	0.08	5/15 vs. 4/24	0.27
Autoimmunity	1/15 vs. 4/20	0.63	3/15 vs. 4/20	>0.99	6/15 vs. 7/24	0.51
Inflammation	0/15 vs. 3/21	0.27	3/15 vs. 4/20	>0.99	5/15 vs. 4/20	0.27
Cytopenia	1/15 vs. 16/24	0.0002	11/15 vs. 19/24	0.71	14/15 vs. 23/24	>0.99
Lymphoproliferation	1/15 vs. 0/24	0.38	3/15 vs. 2/24	0.35	3/15 vs. 2/24	0.35
Treatment					6/15 vs. 7/24	0.51
Molecular diagnosis					5/15 vs. 6/24	0.73
IEI diagnosis					4/15 vs. 2/24	0.18
IgM concentrations over time						0.17
Chronic					5/15 vs. 14/24	
Progressive					4/15 vs. 2/24	
Intermittent					5/15 vs. 4/24	
Resolved					1/15 vs. 4/24	

## Data Availability

The original contributions presented in this study are included in the article. Further inquiries can be directed to the corresponding author.

## Supplementary Materials

The following supporting information can be downloaded at https://www.mdpi.com/article/10.3390/ijms26189003/s1.

## Abbreviations

ESID	European Society for Immunodeficiencies
IEIs	Inborn errors of immunity
IgMD	IgM deficiency
IUIS	International Union of Immunological Societies
sIgMD	Selective IgM Deficiency
